# *“I Was Relieved to Know That My Baby Was Safe”*: Women’s Attitudes and Perceptions on Using a New Electronic Fetal Heart Rate Monitor during Labor in Tanzania

**DOI:** 10.3390/ijerph15020302

**Published:** 2018-02-09

**Authors:** Sara Rivenes Lafontan, Johanne Sundby, Hege L. Ersdal, Muzdalifat Abeid, Hussein L. Kidanto, Columba K. Mbekenga

**Affiliations:** 1 Institute of Health and Society, Faculty of Medicine, University of Oslo, Forskningsveien 3A, 0373 Oslo, Norway; johanne.sundby@medisin.uio.no; 2 Department of Anesthesiology and Intensive Care, University of Stavanger, 4036 Stavanger, Norway; hege.ersdal@safer.net; 3 Temeke Regional Referral Hospital, Dar es Salaam, Tanzania; amuzdalifat29@gmail.com; 4 Ministry of Health Community Development Gender Elderly and Children, Dodoma, Tanzania; hkidanto@gmail.com; 5 School of Nursing and Midwifery, Aga Khan University, Dar es Salaam, Tanzania; kokumbekenga@gmail.com

**Keywords:** Tanzania, low-resource setting, labor care, laboring women’s attitudes, (electronic) fetal heart rate monitoring, labor monitoring, health literacy, informed consent, Moyo, wireless fetal heart rate monitor

## Abstract

To increase labor monitoring and prevent neonatal morbidity and mortality, a new wireless, strap-on electronic fetal heart rate monitor called Moyo was introduced in Tanzania in 2016. As part of the ongoing evaluation of the introduction of the monitor, the aim of this study was to explore the attitudes and perceptions of women who had worn the monitor continuously during their most recent delivery and perceptions about how it affected care. This knowledge is important to identify barriers towards adaptation in order to introduce new technology more effectively. We carried out 20 semi-structured individual interviews post-labor at two hospitals in Tanzania. A thematic content analysis was used to analyze the data. Our results indicated that the use of the monitor positively affected the women’s birth experience. It provided much-needed reassurance about the wellbeing of the child. The women considered that wearing Moyo improved care due to an increase in communication and attention from birth attendants. However, the women did not fully understand the purpose and function of the device and overestimated its capabilities. This highlights the need to improve how and when information is conveyed to women in labor.

## 1. Introduction

While there have been global improvements in child survival, perinatal mortality remains nearly unchanged [1]. Each year, as many as 2 million babies die during labor (fresh stillbirths) [2,3,4,5] and almost 3 million newborn babies die within their first month of life (neonatal deaths). The global target for reducing neonatal mortality as stated by the Sustainable Development Goal 3.2 aims to reduce neonatal mortality to 12 per 1000 live births by 2030 [6]. The countries in the world with the highest neonatal mortality are located in South Asia and Sub-Saharan Africa [7]. While Tanzania has made great improvements in reducing neonatal mortality, 27% of the estimated 8000 newborn deaths occurring each year in the country are caused by birth asphyxia [7]. Birth asphyxia can be detected through regular fetal heart rate monitoring (FHRM). The most common way to monitor FHR is by using a Pinard fetoscope. However, in low-income settings where there is a lack of skilled birth attendants, such monitoring is often not done according to guidelines [8], partly due to time constraints [9]. FHRM has also been found to be suboptimal as the partogram used for monitoring and documenting the progress of labor through regular FHRM and maternal assessment is considered to be a complex tool [10]. While it provides guidance for obstetric interventions based on the progress of labor, it is often under-utilized or incorrectly completed [11,12]. 

To improve FHRM, a new strap-on automatic fetal heart rate monitor, Moyo, was developed by Laerdal Global Health (see Appendix A). It helps detect fetal heart rate and alerts the skilled birth attendant in an effort to ensure timely obstetrical actions and prevent birth asphyxia and fresh stillbirths. Acceptance by users is essential for the success of technological devices [8,13]. While investigated in high-income countries, there is limited knowledge about laboring women’s views about new technological devices used in maternal care in low-resource settings. We believe it is important to bring forward their perspectives in an effort to improve care. This knowledge is important to identify potential barriers towards adaptation in order to introduce new technology more effectively and ensure long-term use. Through a review of literature, we were unable to identify other studies that investigated laboring women’s attitudes and perceptions about a wireless strap-on electronic fetal heart rate monitor in low-resource settings. Research is therefore needed as new technological devices are increasingly introduced in maternal care in low-resource settings. The objective of this present study is to explore the attitudes and perceptions of mothers who wore Moyo during their most recent delivery about the device and its effects on the care they received. 

This study is part of the ongoing evaluation of the introduction of Moyo and was conducted in parallel with the quantitative Safer Births Moyo studies in Dar es Salaam. At a tertiary health facility in the city, a 2-arm randomized control study testing the use of Moyo versus a hand-held Doppler for fetal heart rate monitoring was conducted. At a municipal referral hospital, a descriptive study evaluating the use of Moyo and its effects on timely obstetrical actions/referrals and perinatal outcome was carried out.

## 2. Materials and Methods 

### 2.1. Study Design and Data Collection

As the current study aimed to explore the attitudes and perceptions of laboring women, a qualitative approach was chosen [14]. In order to capture individual experiences, a total of 20 semi-structured individual interviews were carried out [15], ten (10) at each study site. An interview guide was used which included open-ended questions about the information received about the device, opinions about wearing it, and the care received while wearing the device. When necessary, follow-up questions were asked for elaborations or clarifications. Each interview ended by asking the participant if she had any questions for the interviewer. Interviews at both hospitals were conducted in Kiswahili by a research assistant who was a teacher in midwifery with experience in conducting qualitative research. The first author (Sara Rivenes Lafontan) was present during all interviews. Data collection continued until saturation and no new themes arose [15]. Additional interviews were consequently carried out at both study sites in an effort to validate findings with new respondents. This process aims to verify the collected data in order to increase the validity of the findings and is often referred to as respondent validation or member checking [15]. These interviews were part of the total number of interviews carried out. The interviews were conducted 12–24 h post labor at different private locations inside both hospitals to ensure privacy, and lasted 20–25 min. The data collection took place from January to March 2017.

### 2.2. Recruitment of Participants and Ethics

Twenty mothers were recruited to participate in the study and all participants were interviewed once. Recruitment was done through convenience sampling [15]. The mothers were approached before discharge from the post-natal ward and informed about the study by two members of the research team at both hospitals. All the women who were asked to participate in the study accepted. The recruitment was conducted by the Tanzanian research assistant with assistance from nursing staff at the maternity wards. The inclusion criteria to participate in the study were that Moyo had been used during the most recent delivery, that there had been a positive fetal outcome, and that the women were multiparous.

The study was conducted according to the Declaration of Helsinki [16]. All participants received oral and written information about the purpose of the study before giving their written consent to participate. The Safer Births studies are approved by the Norwegian Regional Ethics Committee (REK Vest; Ref: 2013/110/REK vest) and the Tanzanian National Institute for Medical Research (Ref: NIMR/HQ/R.8a/Vol.IX/388). The first author obtained a research permit to carry out the study from the Tanzania Commission for Science and Technology, COSTECH, (No. 2016-396-NA-2016-277). The study obtained ethical approval from all relevant entities, both at the institutions where the study was carried out and at the local government.

### 2.3. Study Setting

The study was carried out at two hospitals in Dar es Salaam, Tanzania. Hospital 1 is a tertiary referral hospital with 10,000 annual deliveries. It receives patients referred from both public and private practice and also serves paying private patients. The obstetric department is staffed with a number of obstetric and gynecologic (Ob-Gyn) specialists, resident doctors, intern doctors, and nurses/midwives. The labor ward includes 19 beds and five birth attendants per shift. Hospital 2 is a municipal referral hospital receiving patients from health centers and peripheral hospitals in a primarily high-density area of Dar es Salaam. There are two Ob-Gyn specialists per day working in the obstetrics department in addition to medical doctors, intern doctors, and nurses/midwives. The hospital has approximately 17,000 annual deliveries, between 40 and 50 each day. The labor ward has 12 beds and five birth attendants during the day. Women at the two facilities were monitored using a Pinard prior to the introduction of Moyo (as a trial). Both facilities have the capacity to perform what is described as comprehensive emergency obstetric and newborn care signal functions [17]. 

### 2.4. Data Analysis

The interviews were recorded and transcribed verbatim by a transcriber who was trained by the first author (Sara Rivenes Lafontan) and who had previous experience transcribing qualitative interviews in Kiswahili. The transcripts were translated into English by a native speaker fluent in both Kiswahili and English and familiar with the study context. Both transcripts and translated versions of the interviews were verified by members of the research team. The translated interviews were read and re-read to deepen the familiarity with the content. Data organization was undertaken using the software package NVivo 11 (QSR International Pty Ltd., Melbourne, Australia). The data was analyzed using qualitative content analysis which is considered suitable for descriptive research questions [18]. During this stepwise process, the material was systematically divided into codes and categories as described by Graneheim and Lundman [19,20]. Transcripts were analyzed line by line and assigned to relevant codes. A coding list was generated and codes were subsequently merged into categories; see Table 1 below for an example of the coding process. Throughout this process, emphasis was on keeping the original wording of the mothers participating in the study. Condensed meaning units, codes, and categories were discussed and agreed upon among the authors. 

## 3. Results

### 3.1. Demographic Characteristics 

The age range of participants was 23–43 years, median age 32 years. A summary of participant characteristics by age group, occupation, and number of children is presented in Table 2 below. 

### 3.2. Categories

The attitudes and perceptions of the women participating in the study towards using the device and their perceptions about how the use affected care were divided into four categories: understanding Moyo’s purpose and functions, feeling the device had a positive effect on the delivery, receiving close care, and feeling good knowing the baby was safe. An additional category was developed to capture the women’s suggestions for how the introduction of Moyo could be improved.

#### 3.2.1. Understanding Moyo’s Purpose and Functions

Half of the participants at Hospital 2 and one participant at Hospital 1 responded that they had not been informed about the purpose of the device and its main functions when it was put on them. All but one of the participants who responded that they had not been informed had asked the health care provider what it was or understood it themselves. This was the only category where there was a clear difference in the responses at the two study sites. Of those who reported that they were informed, the information received and/or retained by the participants seemed to be related to the purpose of the device and less about its functions; they knew that Moyo measured fetal heart rate (purpose), but were unaware of the meaning of the colors on the display and sounds coming from the monitor (functions). None of the participants seemed to have fully understood the functions of the device, including the alarm function. One woman was unable to see the monitor because it was hung on the IV drip stand with the display away from her:

I would like if they could turn the device around so I am able to see and know what’s going on, also if they could give us more information about the meaning of colors and what to do if anything ever happens.

Hospital 1#3

As an explanation for why they did not know certain functions of Moyo or the purpose of the device, six women said that they were unable to absorb information or ask questions about Moyo due to labor pains. One participant indicated that while she had been informed about what the device measured, she had not been informed about its functions but she trusted the health care providers to take the appropriate action if needed. Those who said they had not received information about the purpose of the device did not express more negative attitudes towards the device or about wearing it. However, they more frequently attributed functions to the device; one woman who reported that she had not been initially informed suggested it might be a form of lucky charm since it was worn around her neck. Another thought it was a clock because she saw numbers on the monitor’s display. There was also a tendency by some to overestimate the diagnostic power of the device; one woman said she thought that fetal abnormalities would be detected faster when the device was used. Some of the participants also mentioned that they believed the device helped the baby breathe and helped the baby overall to ensure a safe delivery.

One woman at Hospital 2, who also responded that she had not received information, explained that the woman lying in the bed next to her had said that if Moyo did not make a sound it meant that the fetus was dead. Another also expressed fear of the consequences of the device not making a sound:

In my mind I was thinking maybe if the device did not produce any sound my baby was no longer alive. So from time to time I pulled the straps of the device and waited for the sound.

Hospital 2#20

#### 3.2.2. Feeling the Device Had a Positive Effect on the Delivery

All participants in the study delivered vaginally and on term without major complications during their most recent delivery. The women expressed that wearing the device had positive effects during the delivery. Three of the participants at Hospital 2 mentioned that Moyo helped the labor progress due to the belt which some said held the abdomen up, while another said helped the baby progress through the birth canal:

Previously when pushing the baby after some time the baby returned inside the womb and I had to push again and again. But this time with the device when pushing the baby did not return inside because there were no room for returning, the device had occupied the remaining space.

Hospital 2#16

Despite it being a new device, none of the participants expressed any doubt about the accuracy or safety of the device. For some of the women, the use of Moyo seemed to be linked with medical advancement and improvements in care which translated into an easier delivery for the women:

A high number of women lost their babies but now when the labor pains start when you attempt to push, the baby arrives with little hustle not like in the past when you would be in labor for six to eight hours.

Hospital 1#7

None of the participants said the device was painful to wear compared to the Pinard which some said was painful when it was pressed on the abdomen. Some of the women also expressed feeling that Moyo had given them strength and energy; they had felt less tired and Moyo gave them the strength to push during contractions. There were some conflicting opinions about the effect of Moyo on labor pains. Some participants wondered if wearing Moyo resulted in more labor pain as the pain had become more intense when Moyo was put on. One mother felt that Moyo had contributed to less labor pain. The issue of labor pain and its effects was raised by the mothers and was not part of the interview guide. 

#### 3.2.3. Feeling Good Knowing the Baby Was Safe 

Several of the mothers at both study sites reported previous negative experiences in childbirth, some having lost a child. Many explained being worried about the wellbeing of the baby and receiving limited information about the progress of the baby during previous deliveries:

I lost a child 2 years ago—they found out that one of the babies I carried died and I only found out after I gave birth to the other baby.

Hospital 2#13

This was compared to the feeling of reassurance about the wellbeing of their unborn child when Moyo was used. The continuous signs from the monitor that the baby was doing well, and being able to hear the heartbeats from the monitor and see the FHR marked on the display enabled the women to experience for themselves that the baby was doing well. One woman, when asked what was different during this delivery compared to previous ones, said:

I: Did you feel anything different?

R: Yes, I felt the difference, the difference is this time I could see how my baby was progressing while I was going through labor, the device gave me hope that the baby was ok.

Hospital 1#9

The main focus for the women interviewed was how Moyo positively affected their unborn child and not about how the women themselves felt about wearing the device. Questions about particular features of the device were often answered with the benefits of using the device for the fetus. When asked what it felt like to wear Moyo during the delivery, one woman simply responded:

I was relieved to know that my baby was safe.

Hospital 2#7

#### 3.2.4. Receiving Close Care 

The use of the device seemed to increase the sense of receiving care and being monitored for many of the women in the study. Some of the participants said they felt they had received closer follow-up from the health care provider compared to previous deliveries and said that even if the nurse/midwife was not by the bedside, she was monitoring the progress of the delivery from afar:

Respondent: even though the midwife was away she was able to hear.

Interviewer: she listening when away?

Respondent: Yes.

Hospital 2#7

When comparing Moyo to the Pinard, the increased monitoring was something that was pointed out by some of the women:

I think there’s more care and attention given when Moyo device was used, they’d attach it from the beginning until you give birth and they’d monitor it in between whereas with Pinard, they’d only monitor once in a while—when you are first admitted and when you are giving birth.

Hospital 1#8

Moreover, two participants said that they felt they had received more attention from the health care provider when Moyo was used. One of these said that despite receiving less attention, she felt reassured about the progress of her child because she could see it on the device. It could seem as though the use of Moyo gave the health care providers more reason to attend to the mother if only to check on the device. Participants explained how the midwives came to look at the display of the device and left again without taking any other measurements or observations. 

When Moyo was used, the mothers felt more actively engaged in the labor monitoring process which they also expressed as positive. Several respondents described how the monitoring of the fetus became a shared responsibility between the mother and the health care providers because the mother could follow the fetal heart rate. One participant said she felt there was an increased collaboration between her, the doctor, and the midwife.

I: So how did you feel when you saw that your baby was ok?

R: I felt more confident... there was also a lot of cooperation around, compared to the first device (Pinard).

I: Why was there no cooperation when the first device was used?

R: Because only a doctor/nurse could hear.

Hospital 2#11

#### 3.2.5. Suggestions for Improvements

None of the participants in the study had suggestions for how the functions and characteristics of the device could be improved. However, it was suggested that Moyo should be introduced during ante-natal care (ANC) visits in order for the mothers to receive adequate information and have time to familiarize themselves with the device before arriving at the labor ward.

I would suggest that the patient is educated about the device before coming into the labor ward, we are often in so much pain when we enter the (labor) ward, so it’s not easy to listen and take everything in, some may refuse to wear the device because they are worried or in doubt and don’t want to add more pain, so it’s best that patients are told about the device before they enter the ward.

Hospital 1#8

Others said that their only suggestion was that Moyo should be available to as many women as possible during labor. It was explained that it would benefit both women and their babies, making the childbirth easier for the women.

## 4. Discussion

In the present study, we explored the attitudes and perceptions of women using a new electronic fetal heart rate monitor during labor. Our results indicate that the use of the monitor positively affected the women’s birth experience by providing much-needed reassurance about the wellbeing of the child. The mothers also believed that the care had improved due to a perceived increase in communication and attention from the health care providers, but also to what the women described as being “monitored from afar”.

Expressing that they were being monitored while the health care provider was away suggests that the women felt monitored due to the fact that they were wearing the device. As such, wearing the device became an extended part of the care provided by the birth attendant. Central to perceptions about care is the presence of the provider and by wearing the device the women expressed increased satisfaction with the care received [21]. However, it has been found that women are positive towards any intervention received during ANC or labor, regardless of the efficacy of the intervention [22]. The fact that many expressed that they felt care improved with the use of Moyo could also have to do with possible neglect experienced in the past [23]. Expressed satisfaction with the care received could also be an indication of low expectations, or not knowing what to expect [24,25]. Often, during labor and delivery, women with low socio-economic status in overburdened public facilities are seemingly quite powerless, passive, and poorly informed, and have low expectations about care and information [26,27]. One could also argue that it might be difficult for the women to judge the quality of care without having experienced good care in the past. Several of the women in the study expressed receiving limited information and labor monitoring during previous deliveries, which could be another reason why the perceptions about care were mainly positive. Studies indicate that receiving medicines or items such as bed nets is described by women as good care while not receiving information from health care providers was not associated with poor care [24].

The reported lack of information by some of the participants about the purpose and functions of Moyo seemed to generate misconceptions and an overestimation of the capabilities of the device. The device was considered by some as almost magical in its abilities and some participants believed that Moyo not only detected but also solved problems by helping the baby to breathe or giving the mother the strength to push during delivery. This finding is similar to a qualitative study about the use of ultrasound in antenatal care in Botswana [28]. The women who reported that they were not informed more frequently reported attributions and an overestimation of the capabilities the device. This indicates an unmet need for information about Moyo and draws on models of health literacy and informed consent. These concepts imply that the patient receives and understands information about purpose, limitations, and procedure and the choice to accept or decline prior to a medical procedure [23]. To increase people’s health literacy is an international priority as low health literacy is linked to increased morbidity and mortality [29]. Health literacy is also a critical component of empowerment as limited health literacy reduces autonomy in self-care and decision-making [30]. 

For the women in our study, the use of Moyo seemed to have strengthened their position during the delivery and the device became a tool of empowerment. In low-income settings, women are perceived as having less access to essential resources and less autonomy and decision-making power compared to men according to studies [31]. Each year, roughly a third of maternal deaths worldwide are directly related to inadequate care during pregnancy [32]. Conversely, empowered women have lower infant mortality and better overall health [31,33]. By wearing the device and monitoring the FHR, the women took on a more active role as they themselves were part of the important task of monitoring the progress of their baby. The combined effect of knowing the status of their unborn child and what they perceived as increased attention from the health care providers created a feeling of confidence, particularly among the participants in the study with the lowest socio-economic status. This contribution to the empowerment of the women in the study is an aspect of technology diffusion in low-income settings that we believe should be investigated further. 

To measure FHR, many of the women in the current study preferred Moyo compared to the Pinard fetoscope and did not express concern about Moyo being a new device. It is argued that women have more confidence in information produced by technological devices rather than in their own bodily sensations as technology is often associated with experts and valued over local practices and the intervention-free birth which is perceived as “risky” [28,34,35]. This phenomenon is described as Gizmo idolatry, defined as *the willingness to accept, in fact to prefer, unproven, technologically-oriented medical measures* and that machinery is considered more valuable than a “low-tech” approach [36]. This attitude could explain why none of the participants expressed any fears about the potential harm of using the device which was a surprising finding and contrary to previous studies on the use of ultrasound [23,28]. 

Many of the respondents expressed a sense of relief knowing that their child was doing well when using Moyo. FHRM seemed to be considered a test to find out if everything was okay, compared to a confirmation that it was. This finding is similar to other studies in Sub-Saharan Africa investigating attitudes toward the use of ultrasound during pregnancy [37]. The anxieties of childbirth, particularly pertaining to uncertainties about the wellbeing of the unborn child, had been largely ignored by health providers during previous deliveries. The need for reassurance due to the risks involved in pregnancy and childbirth for mothers in low-resource settings is closely linked to the need for information about the labor progress. The fact that some of the women who said they had not been informed either guessed or asked the health care provider about the purpose of the device also indicates a need for control over the labor process, not solely relying on the expertise of the health care providers. Studies from Tanzania indicate that women during ANC and labor receive inadequate information about the status of the fetus and indications, process, and results of medical interventions [23,38]. However, it is argued that most patients are unable to recall information provided to them [39]. As mentioned by some of the women, labor pain makes it difficult to absorb information and the women would most likely have been more susceptible to retaining information provided at an earlier stage of the labor when they were in less pain. 

### Strengths and Limitations

Several steps were taken to increase the validity of the study findings and ensure trustworthiness [20,40,41]. In an effort to increase credibility by shedding light on the research question from different angles, participants in the study varied in socio-economic background, occupation and age. As interviews were conducted in Kiswahili and translated to English, there was a risk that meaning might be lost during the translation process. Translations were therefore verified by members of the research team and the findings were validated with new participants after saturation was reached, in an effort to ensure that concepts were accurately captured. During the data collection, analysis codes were shared, discussed, and agreed upon among authors. The research team was multi-professional with both Tanzanian and Norwegian members, which facilitated interpretation of the data from different angles in order to capture diverse perspectives on the findings. Qualitative findings cannot be generalized due to small and demographically non-representative sample size; however, by describing in detail the context and characteristics of the participants in the current study, we allow the reader to make an informed decision about the transferability of study findings to other contexts [41]. While the women in the study seemed at ease during the interview, they might have felt uncomfortable saying anything negative about the care due to fears of repercussions as they were still admitted to the hospital. A suggestion for future studies is therefore to broaden the group of participants and to interview participants outside of the health care facilities. The women in the current study did not report experiencing severe complications during the most recent delivery and often described it as faster or less painful than previous deliveries. Overall, women with uncomplicated deliveries without unexpected levels of pain and duration of the labor report higher levels of satisfaction with care compared to those who do experience complications. This might be one of the reasons the responses were largely positive, both about the device and about the care received [21,42,43]. 

## 5. Conclusions

This study provides an understanding of how the use of a new electronic fetal heart rate monitor had a positive effect on the birth experience of the women in our study. This was largely due to an increased knowledge about the wellbeing of the unborn child and a perceived improvement in care. The study highlights the unacknowledged anxiety of childbirth which should be addressed by both health care providers and policy makers. A lack of understanding of the basic functions and purpose of the device raises the issue of informed consent and health literacy and the need to improve how and when information is conveyed to women in labor. We recommend that information about new devices used in the labor ward is included in the information provided to pregnant women during ante natal care and/or provided in the early stages of labor. This information should also include limitations of a technological device to avoid overestimation of the diagnostic power. 

## Figures and Tables

**Table 1:** Example of the analysis process.

Translated Transcribed Interview	Code	Category
I: Okay, great, so can you tell us if this device changed your birth experience compared to your previous deliveries where devices like Pinard were used? R: Yes, I saw the difference because this device allowed the nurse to be closer as opposed to previously where they’d walk around and monitor from afar, they would come to me more often too.	Feels that she received closer and more frequent attention from the nurse compared to previous deliveries due to the device.	Receiving close care ^1^

^1^ The category was formed by several codes.

**Table 2:** Demographic description of participants by age group, occupation, and number of children.

Variable	Sub-Groups	*n* (20)	%
Age	20–29	6	30
	30–40	13	65
	above 40	1	5
Occupation	Run a small business	7	35
	Maid	1	5
	Teacher	2	10
	Stay at home	5	25
	Farmer	2	10
	Nurse	1	5
	Entrepreneur	1	5
	Business woman	1	5
Number of children	1		
	2	6	30
	3	4	20
	4	8	40
	above 4	1	5

**Figure A1:**
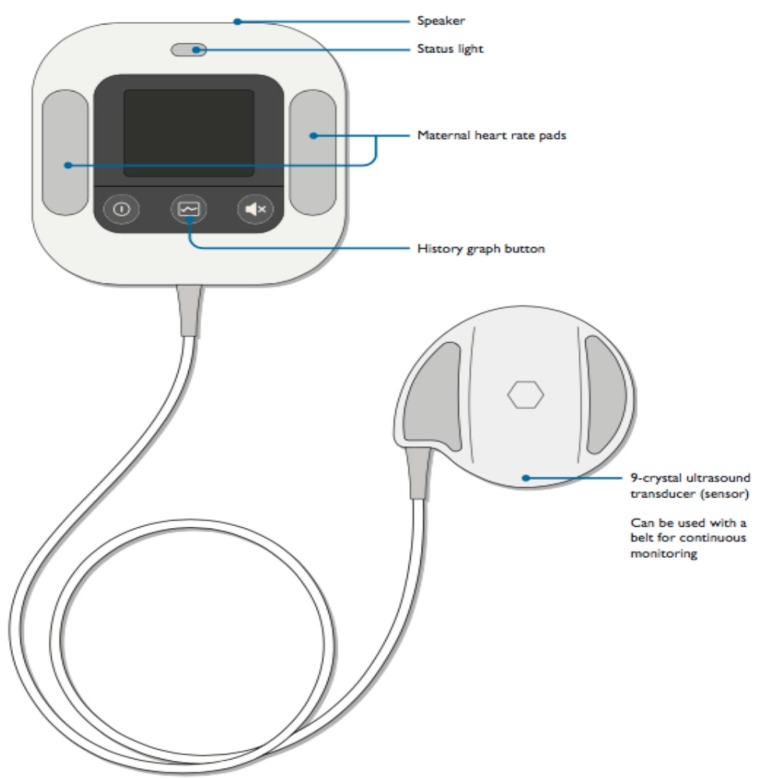
The Fetal Heart Rate (FHR) monitor, Moyo (Laerdal Global Health).
